# At the Same Table: A Delphi Consensus-Based Model of Health-Promoting Eating Behaviors

**DOI:** 10.3390/nu15163601

**Published:** 2023-08-17

**Authors:** Rachel F. Rodgers, Valerie Goutama, Kendrin Sonneville

**Affiliations:** 1 APPEAR, Department of Applied Psychology, Northeastern University, Boston, MA 02115, USA; 2 Department of Psychiatric Emergency & Acute Care, Lapeyronie Hospital, CHRU Montpellier, 34295 Montpellier, France; 3 Department of Nutritional Sciences, University of Michigan School of Public Health, Ann Arbor, MI 48109, USA; kendrins@umich.edu

**Keywords:** Delphi, panel, health, diet, nutrition, expert, guidelines

## Abstract

Eating behaviors and patterns are one of the key behavioral indicators of health, and as such these behaviors are a focus of interest across different areas of scholarship. Yet, to date, work in this area is impeded by the lack of a collective theoretical framework to conceptualize, assess, and intervene upon eating behaviors. The aim of this study was therefore to establish a consensus-based framework for health-promoting eating behaviors using a Delphi methodology. An initial systematic search identified constructs that yielded 150 items grouped into three topic areas: (1) the content, types of food and nutrition provided; (2) eating behaviors; and (3) thoughts and feelings related to eating and foods. Over the course of three iterative rounds of rating by a panel of *n* = 37 experts, a consensus was reached that included eight of the original items that represented the three topic areas. The findings from this study result in a novel consensus-based framework for health-promoting eating behaviors that can form the basis for collaborative work towards the integration of physical and mental health promotion.

## 1. Introduction

Eating behaviors and diet are now well-established as one of the principal contributors to positive physical and mental health status and lower mortality [1]. Indeed, about half of all U.S. adults, approximately 117 million adults, have one or more preventable chronic diseases largely related to eating patterns [2], which disproportionately includes underserved and marginalized individuals [3]. Thus, health-promoting eating patterns have emerged as a primary target for public health. However, overall, efforts to improve eating have been met with a lack of success [4,5]. This lack of success is likely in part due to the narrow and somewhat reductionistic conceptualization of “healthy” eating that has prevailed, as well as differences across areas of scholarship regarding the conceptualization and definition of eating patterns and behaviors that would be helpful for individuals [6,7]. These divergent viewpoints constitute barriers towards moving towards the development of useful conceptual frameworks that can be used to inform effective interventions.

The predominant definition of healthy eating relies on an energy balance model of weight maintenance that targets controlled caloric intake (portion size, lower caloric foods) and energy expenditure (mainly through physical activity) [8]. The energy balance model understands the benefits of a balanced intake to be largely attributable to indirect relationships via weight control. Nevertheless, the energy balance model places emphasis on foods themselves and neglects other factors (emotions, values, etc.), as well as tending to attribute the quality of “healthy” or “unhealthy” to specific foods. As outlined by the Academy of Nutrition and Dietetics, such labeling promotes dichotomous thinking, denies the complementary nutritional values of foods, and may paradoxically lead to poorer eating outcomes due to the need to restrict “bad” or “forbidden” foods [9]. In addition to the energy balance model, other understandings based on nutritional content and patterns have emerged. These models consider patterns as including the amounts, proportions, combinations or varieties of different foods or nutrients and their frequency [6,10]. High-quality food patterns are believed to confer benefits for physical health including promoting cardiovascular health, preventing diabetes, and reducing inflammation to be due to the interactive nature of the nutrients consumed over time [11]. While this is a less reductionistic understanding compared to the energy balance model, it also fails to account for any of the social, cognitive, and emotional context of eating behaviors. It should also be noted that both of these models are limited in their consideration of food justice and ill-suited to situations in which food insecurity or poor food accessibility are present.

Alternative models of healthy eating that include other dimensions have recently emerged, notably the intuitive eating [12,13], the mindful eating models [14], as well as the eating competence model [15]. Although the evidence is less robust, all three of these eating patterns have been suggested to be associated with physical health benefits including greater physical activity, lower cholesterol and blood pressure, or lower risk of diabetes [16,17,18]. Importantly, they have also been shown to be associated with psychological health benefits including improved psychosocial functioning and lower levels of disordered eating [12,14,18]. Intuitive and mindful eating frameworks posit that the benefits derived stem from improved mindful awareness and bodily connection, as well as increased focus on attunement and responsiveness to physical needs [12,13]. Eating competence includes considerations of contextual skills as well as acceptance and flexibility relating to food, eating pleasure, and satisfaction [15]. In contrast to the models described previously, these place an exclusive focus on regulation, and the thoughts and feelings surrounding eating, and neglect any diet-related indicators. Thus, they also fail to capture important aspects of healthy eating such as diet quality, and do not have an explicit focus on vulnerable groups (e.g., those with food insecurity) or the ways in which the current food environment is designed to disrupt intuitive and mindful eating processes.

Thus, the existing models of health-sustaining eating behaviors are limited by their respective narrow focus on certain aspects of eating with either accounting for nutrient content and energy density on the one hand, or solely psychological context on the other. These very separate conceptualizations and different understandings of the mechanisms of action, in addition to different outcomes of interest constitute a barrier to common concerted efforts to better characterize holistic health-promoting eating behaviors and identify modifiable targets with a view to improving intervention efforts. 

Moreover, an important weakness of all these frameworks is their failure to explicitly consider the perspectives, needs, and values of groups of people who have been framed as “at risk” in terms of healthy eating. These groups may include those experiencing food insecurity, low food accessibility, oppression, or disability, or those who have been engaging in chronic rigid and restrictive eating patterns due to the Western abundant food environment coupled with unattainable appearance ideals [19,20]. While “at risk” groups are often the targets of behavioral interventions focused on eating behaviors [21], there is often insufficient consideration of the unique needs of such groups in the design and delivery of the interventions. An additional complicating factor relates to the extent to which such interventions are disproportionately directed at individuals in larger bodies and utilized as a conduit for weight control, potentially limiting their utility and reinforcing weight bias [22]. As such, our goal is to move towards a definition of healthy eating that is independent of body size.

## 2. Method

### 2.1. Study Design

The current study utilizes the Delphi method by inviting experts across different fields to make independent ratings on a series of statements across several rounds. These ratings were then used to produce a final set of statements that represent a consensus from the group. The process utilized four steps, as previously described [23]. The first step consisted in identifying a corpus of statements to be rated, and the second in identifying a panel of experts. Third, expert feedback was sought over three iterative rounds for ratings of each item. Finally, the results of these successive rounds of ratings were utilized to create the final consensus product. 

### 2.2. Participants

Given the observation that discipline-specific conceptualizations and methods were among the major barriers to advancement in this area, experts were sought from a range of areas of scholarship (eating disorders, obesity, food justice, etc.) as well as specializations and trainings (psychologist, dietitians, public health, etc.). In addition, efforts were made to maximize the diversity of the expert pool in terms of gender, seniority, race/ethnicity, and other identities. Expertise was determined through involvement in research and scholarship and/or clinical practice, the development of previous measures of eating behaviors, and the participation in the development of relevant guidelines or reports. Experts were contacted via email containing information regarding the study goals and procedures as well as a participant information sheet. The goal was to recruit approximately 30 panel experts to allow for diversity and in anticipation of attrition across the study rounds [23,24]. Indeed, comparable studies in the field have included similar sample sizes ranging from *n* = 26 to *n* = 47 [24,25,26]

An initial group of 30 expert participants was contacted to complete the first round of the survey. Given the low response rate (30%, *n* = 9), a second group of 44 additional experts was identified and contacted. The second pool of experts was identified with particular concern to diversity as well as more personalized invitations. Together, these efforts yielded a total of 37 experts who participated in the first round of the study. The majority of participants were female (86%) who identified as White (62%), while others identified as African American/Black (17%), Hispanic (12%), and Asian (4%). Some participants reported more than one racial/ethnic background. Participants were aged on average 53.7 years old (*SD* = 7.88). A total of 24 experts responded to the Round 2 survey (64% of Round 1). The third and final round survey was completed by 23 experts (95% of Round 2).

Participants indicated their primary field of expertise from a list of five options: Eating Disorders (*n* = 13, 35.1%), Nutrition (*n* = 8, 21.6%), Obesity and Overweight (*n* = 3, 8.1%), Public health, Governmental Guidelines or Programs (*n* = 2, 5.4%), Weight Stigma or Health Disparities (*n* = 6, 16.2%), or Other (*n* = 5, 13.5%). The 5 individuals reporting “Other” specified their backgrounds, which included professions such as Eating and Dieting Behavior and Epidemiology. Table 1 summarizes the participants’ primary fields.

### 2.3. Items

Statements were generated through a systematic search of resources related to healthy eating. Specifically, three online search engines (Google US, Google UK, and Google Australia) were searched for key terms, and the first 20 sites for each term on each search engine were recorded. The search terms included: healthy diet, healthy eating, healthy eating behavior, and healthy nutrition. Duplicate websites were removed throughout the process. Relevant books and publications by expert authors were also located via references or knowledge. The search generated a list of concepts and statements that were then utilized to generate the pool of items to be rated by the expert panel participants. 

Following previous methods [24], two members of the research team read each of the websites along with the additional materials and extracted unique ideas related to healthy eating. These ideas were listed and any duplicate ideas were removed. The final list was then subject to content analysis by four members of the research team. As previously [24,27], broad themes were first developed, and ideas were grouped within these. Once a structure had been identified, statements were then drafted to capture each idea. The statements were all phrased to illustrate elements that might be judged to be representative of “healthy” eating. For example, “Eating a balanced diet”, or “Cooking with oil”. 

This process rendered an initial pool of 150 statements to be rated in the first survey, that were organized into three overarching themes. The first included items focused on the content, types of food, and nutrition provided. The second theme included items focused on eating behaviors, and the third theme included items focused on thoughts and feelings related to eating and foods. In the first round of ratings, participants were invited to provide feedback on the items and suggestions for changes to wording as well as the inclusion of additional concepts. 

### 2.4. Measures

The online survey asked participants to rate each item on a 5-point scale (Essential, Important, Do not know/Depends, Unimportant, Do not include) indicating how important it was to include the item as contributing definition for “healthy eating”. Survey items were rated according to the widely used matrix in previous studies. 

### 2.5. Statistical Analysis

The following criteria were used: If endorsement rate was between 90% to 100%, and most experts agree that the item should be retained, it was included for the following rounds. If endorsement rate was between 80% to 89% percent, experts did not reach a consensus that the item should be retained and it would then be included and re-rated in the following rounds. During the final round, items within that range that did not receive full endorsement were excluded from the final consensus. If endorsement rate was below 79%, then the majority of experts did not endorse the item, and hence would be excluded in the following rounds. At the end of the first round, the expert feedback and suggestions for modifications or additional inclusions was reviewed, and modifications were made for the second round. 

### 2.6. Procedure

The study was approved by the relevant institutional review board. No compensation was provided. Expert panelists were contacted via email with an invitation to participate as well as the link to the first survey. The survey contained a link to a separate sheet in which panelists were invited to provide their email such that they could then receive the link to the second and third rounds of ratings. Thus, responses were anonymous. With the links to the second and third round of ratings, participants received a report of the results from the previous round of ratings at the group level and were invited to use this information in making their subsequent evaluations. At the end of the study, participants received a final report detailing the items that were ultimately retained. 

## 3. Results

**Figure 1:**
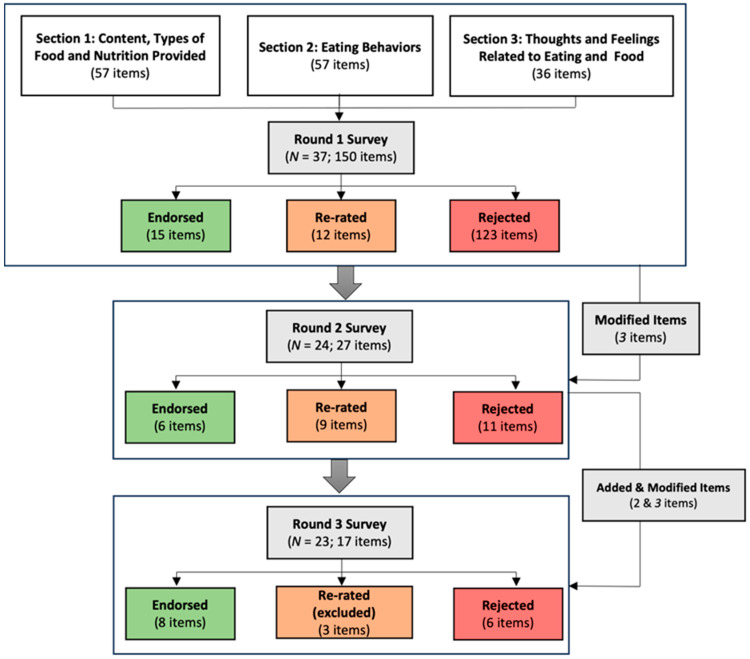
Flowchart illustrating the iterative Delphi process.

The systematic review revealed a variety of sources, including international guidelines (e.g., https://www.who.int/news-room/fact-sheets/detail/healthy-diet) (accessed on 15 October 2020) as well as largely unmoderated forums or opinion blogs focusing on weight and shape control (https://www.nerdfitness.com/blog/healthy-eating/) (accessed on 15 October 2020). Of the 150 items presented to the panel, 123 were endorsed by 79% or fewer of the experts and were therefore not moved forward to the next round of ratings (see Figure 1). Based on qualitative participant feedback collected at the end of the first survey, three items were reworded. Specifically, items that had referred to diet or nutrition “quality” were reworded to avoid use of those terms, and an item referring to water was reworded to refer to broad non-sweetened drinks. Thus, the second survey contained 12 items that had been endorsed between 80–90% to be re-rated, as well as the 15 items that were strongly endorsed. Following the second round of ratings, another 11 items were discarded due to very low endorsement, and 9 were included for re-rating given only partial endorsement (80–90%). Moreover, an additional four items were reworded to remove reference to make items more generalizable, for example “in the kitchen” was replaced with “available”, or to improve the item grammar and readability. Finally, at the end of round 3, another six items received low endorsement, and four received partial endorsement leading them to be discarded, and a final eight were retained (see Table 2). For a full list of each of rejected items, see Appendix A and Appendix B.

## 4. Discussion

Eating behaviors and patterns are associated with short- and long-term physical and psychological health impacts [1]. As such, assessing these behaviors, understanding their determinants at multiple levels, and developing effective interventions to support health-promoting eating behaviors are critical directions. To date, these efforts have been impeded by the lack of a common definition and framework for health-promoting eating behaviors across scholarly disciplines. The aim of this study was to create an interdisciplinary consensus regarding the definition and characterization of healthy eating with the goal of supporting future research and practice. The conceptualization resulting from this consensus reflects this interdisciplinarity by including a focus on the food consumed, as well as the behavioral, cognitive, and emotional aspects of an individual’s eating patterns. As such, the hope is that this model will pave the way towards more collaborative work in the area of eating.

The results of the systematic review conducted to identify the items to be included for rating by the expert panelists led to the identification of three main areas or themes. The first was related to content, types of food and nutrition provided, the second focused on eating behaviors per se, and the third included thoughts and feelings related to food and body image. The inclusion of items from all three of these areas in the final conceptualization highlights their importance as critical elements of eating patterns. In addition, the review led to the identification of 150 initial statements, of which 123 were strongly rejected due to low expert endorsement after the first round of ratings. This is a very large proportion compared to some other studies utilizing similar methodologies in which the majority of items were retained after the first round [26]. Although notably, some Delphi studies focusing on areas of debate related to eating disorders have encountered similar low consensus for many items [28]. It is likely that, in our study, the low consensus in the first round was partly due to the range of areas of expertise held by our panelists leading to very disparate views. Given that it was our explicit aim to identify commonalities in these largely divergent viewpoints, the elimination of so many items in the first round suggests that we were successful in this objective. 

The final endorsed items focused on diet variety across food groups, with specific focus on vegetable products, attunement to inner signs of fullness and satiety, and flexible positive attitudes towards eating. Together, these items represent an understanding of health-promoting eating patterns and behaviors that is inclusive across food groups and accompanied by a versatile and positive relationship to food. This is interesting in its contrast with perhaps popularized conceptions of health-promoting culture and beliefs that may focus on the avoidance or restriction of appetizing and enjoyed foods, in addition to effortful quantified approach to eating according to authoritative guidelines [29,30]. In contrast, the model proposed here revealed the importance of alignment with an inner knowledge of the body’s needs, a responsiveness to those, and a kinder, less regimented approach to food and eating in the context of a varied diet with a focus on plant products [7,8,31]. Such a model is seemingly aligned with aspects of eating that have been shown to be associated with physical and psychological benefits [12,32]. Moreover, plant-based diets have also been highlighted as having a number of benefits in terms of sustainability [33].

One of the guiding concepts throughout the study was the consideration of power and privilege as related to eating patterns and behaviors. It is worth noting that access to a varied, and plant-rich, diet may be inequitably distributed among groups due to financial resources and food scarcity. In addition, barriers to a comfortable relationship with food and reliance on attunement to inner cues may be greater among certain groups due to their current and past experiences of trauma/oppression. In addition, those who experience high levels of preoccupation or concern regarding shape and weight, or difficulty in regulating their eating behaviors according to inner cues may also find such a model difficult to adhere to. Finally, public health messaging that promotes body ideals and eating patterns of the dominant culture reinforce hierarchies and systems of oppression that harm those who are often targeted by public health messaging because of their body size, economic status, or race and ethnicity. 

If this is indeed a consensual model of health-promoting eating patterns and behaviors, implications exist for the framing of public health messaging, the training of professionals across areas of specialization, and the targeting of interventions aiming to support individual behaviors. Greater focus at the public health level should be placed on the development of a comfortable and positive relationship with food. In addition, common curriculum content might be developed for health providers across areas whose work is relevant to eating behaviors to ensure that this more holistic model is promoted. The model also holds implications for policy and practice, including policies relating to the food industry and food environment. It has been noted that the food environment creates specific barriers to eating in the way upheld by this model. In particular, the focus of the food industry can be viewed as discouraging plant-rich diets through the disproportionate development, marketing, and selling of products that are largely not plant-based [34,35]. In addition, the deliberate focus on developing foods that are highly appetizing (as opposed to naturally occurring foods) is in opposition to a model based on promoting attunement to inner cues [36]. It would therefore be important to consider how regulations and practice guidelines could be modified to decrease the environmental barriers to eating in the way suggested by the model proposed here.

The study includes a number of limitations. Despite concerted efforts, the final panel lacked diversity in terms of expert gender as well as race and ethnicity. While this no doubt reflects some of the systemic difficulties we face as a field in terms of increasing diversity within training programs and providers, it may have led to some perspectives being less represented. While the initial survey items that were produced from this study represent a consensus of the expert panelists, they are highly dependent on the expertise of those respondents and the utility of these items as meaningful measures of health-promoting eating behaviors is unknown. The reliability and validity of these items should be tested in future studies. Despite these limitations, a notable strength of this methodology is that is allows experts of diverse professional backgrounds to participate anonymously and individually, which likely enhances their ability to respond truthfully by minimizing the influence of dominant personalities/perspectives.

In conclusion, the findings from this consensus revealed a novel, consensus-based framework for health-promoting eating behaviors that can form the basis for collaborative work towards the physical and mental health promotion. Future work should aim to empirically examine the usefulness of this framework as well as its acceptability among professionals from different areas.

## Figures and Tables

**Table 1:** Primary academic field identified by participants.

Academic Fields	*n*	%
Eating Disorders	13	35.1
Nutrition	8	21.6
Obesity and Overweight	3	8.1
Public Health, Governmental Guidelines or Programs	2	5.4
Weight Stigma or Health Disparities	6	16.2
Other	5	13.5
Total	37	100

**Table 2:** Items with strong consensus in the final round.

Survey Items	% Endorsement
*Section 1: Content, Types of Food and Nutrition Provided*	
1. Eating a variety of vegetables from all subgroups—dark green, red and orange, legumes (beans and peas), starchy	91.3
2. Eating carbohydrates, proteins, fats, and vegetables	91.3
3. Eating a varied and balanced diet	100
*Section 2: Eating Behaviors*	
4. Using signals of hunger, fullness, and satiety to guide eating	91.3
*Section 3: Thought and Feelings Related to Eating and Food*	
5. Avoiding an “all or nothing” approach	100
6. Being positive, comfortable, and flexible with eating	100
7. Eating to maintain overall health and well-being	91.3
8. Focusing on overall eating habits rather than counting calories	91.3

Note. Highly endorsed items had ≥90% the experts rating them as Essential or Important.

## Data Availability

The data are not availability due to privacy concerns.

## Appendix A. Rerated Items Not Reaching Consensus

**Table A1:** Rerated items not reaching consensus in section 1.

Section 1: Content, Types of Food and Nutrition Provided.	% Endorsed
*Round 1*	
1. Prioritizing quality of food over eating low-carb or low-fat	81.2
2. Eating a variety of vegetables from all subgroups—dark green, red and orange, legumes (beans and peas), starchy	84.4
3. Preferring water over sugar sweetened drinks	84.4
*Round 2*	
1. Eating a varied and balanced diet	87.5
2. Eating a variety of vegetables from all subgroups—dark green, red and orange, legumes (beans and peas), starchy	83.3
3. Preferring water over sugar sweetened drinks	87.5
*Round 3*	
No items for this section	

**Table A2:** Rerated items not reaching consensus in section 2.

Section 2: Eating Behaviors	% Endorsed
*Round 1*	
1. Focusing on making pleasurable meals	87.5
2. Listening to and connecting with hunger	87.5
3. Basing eating upon signals of hunger, fullness, and satiety	87.5
4. Eating foods that make your body feel good	84.4
*Round 2*	
1. Basing eating upon signals of hunger, fullness, and satiety	83.3
2. Making time for meals	87.5
3. Keeping healthy foods in the kitchen	83.3
*Round 3*	
1. Making sure that healthy foods are available	86.9
2. Eating nourishing foods that are also satisfying	86.9

**Table A3:** Rerated items not reaching consensus in section 3.

Section 3: Thought and Feelings Related to Eating and Food.	% Endorsed
*Round 1*	
1. Accepting that everyone’s eating experiences are unique	87.1
2. Acknowledging responses to food without judgement	80.7
3. Having relaxed self-trust about managing food and eating	80.7
4. Focusing on the quality of nutrition instead of counting calorie	80.7
5. Acknowledging there is no “good” or “bad” food	80
*Round 2*	
1. Having relaxed self-trust about managing food and eating	83.3
2. Learning to cope with anxiety and guilt about food	83.3
3. Eating foods that are satisfying	87.5
*Round 3*	
1. Respecting your body and not being overly critical	82

Note. Re-rated items had between 80–89% of experts rating them as Essential or Important. Items that fall within this range in the final round will be excluded.

## Appendix B. Highly Rejected Survey Items

**Table A4:** Highly rejected survey items in section 1.

Section 1: Content, Types of Food and Nutrition Provided.	% Endorsed
*Round 1*	
Making sure every meal has carbohydrates, protein, and fat	26.4
2. Eating at least three different items in a meal	25.5
3. Having meals with at least 3 g of fiber from plants, 3 g from carbs, and 3 g of healthy fat	11.7
4. Making sure most of the daily calories are coming from fresh fruits and vegetables, whole grains, legumes, nuts, and lean proteins	58.8
5. Eating mostly foods derived from plants-vegetables, fruits, whole grains, and legumes	52.9
6. Eating one third protein, one third fruit/vegetable, and one third grain/starch	5.8
7. Eating one half vegetables, 1/4 starchy foods, and 1/4 protein	6.1
8. Eating a low-calorie, low-carb, low-sugar, and low-fat diet	5.8
9. Eating the same number of calories you expend	17.6
10. Eating non-starchy vegetables	45.4
11. Eating at least one dark green and one orange vegetable per day	21.2
12. Eating a minimum of 1/2 cup of fruits or vegetables	45.4
13. Having at least three different colors of fruits and vegetables	15.5
14. Eating whole-grain bread instead of refined	58.8
15. Making sure whole grains constitute half of consumed grains	27.2
16. Eating more fiber	78.7
17. Eating one serving of whole grain per meal	6.1
18. Eating snacks that are between 150–250 calories	6.1
19. Eating up to three snacks	3.2
20. Eating healthy and nutritious snacks instead of sweets	60.6
21. Eating natural fats	75.0
22. Incorporating more high-quality fats into the current diet	62.5
23. Incorporating more low-fat protein into the current diet	43.8
24. Eating one serving of a protein-rich food per meal	21.9
25. Eating fat free or low-fat dairy instead of full-fat dairy products	12.5
26. Eating modest portions of meat and dairy	50.0
27. Eating foods with a high water content	15.6
28. Reducing the amount of sodium eaten	74.2
29. Cutting down on processed sugar, animal fat, saturated, and trans fat	78.1
30. Choosing one between bread, wine and dessert	0.0
31. Eating fruits instead of drinking them	53.1
32. Eating fresh berries instead of dried fruit	25.0
33. Taking Omega-3 and Vitamin D supplements	9.4
34. Consuming enough calcium and potassium	68.8
35. Baking or roasting food instead of grilling or frying	28.1
36. Cooking with olive oil	34.4
37. Choosing popcorn instead of chips	3.1
38. Choosing baked potatoes over french fries	25.0
39. Incorporating more antioxidants into the current diet	59.4
40. Incorporating more organic food into the current diet	18.8
41. Consuming foods that increase the body’s metabolism	3.1
42. Eating a vegetarian diet	0.0
43. Eating a Paleo diet	0.0
44. Eating superfoods and fitness foods	3.1
45. Staying away from “diet” foods	53.1
46. Eat low-fat protein	29.0
47. Drinking less alcohol	50.0
48. Varying protein routine	21.9
49. Drinking 100% fruit juice when picking juices	43.8
50. Eating the right amount of calories for you based on your age, sex, height, weight, and physical activity level	50.0
51. Meeting nutritional guidelines	59.4
52. Controlling portion size	50.0
*Round 2*	
Prioritizing quality of food over eating according to a particular diet	70.8
*Round 3*	
Choosing non-sweetened drinks over sugar sweetened drinks	73.9
2. Prioritizing foods that are nutritious over a particular type of diet	69.5

**Table A5:** Highly rejected survey items in section 2.

Section 2: Eating Behaviors	% Endorsed
*Round 1*	
Putting utensils down between bites	12.5
2. Sitting down to eat	65.6
3. Eating slower	50.0
4. Taking small bites	21.9
5. Chewing more often	25.0
6. Chewing thoroughly	43.8
7. Serving your food onto a plate or bowl	53.1
8. Eating from smaller plates	18.8
9. Starting with a small portion	37.5
10. Eating greens first	21.9
11. Eating a big lunch	3.2
12. Having the salad dressing on the side	15.6
13. Focusing on making pleasurable meals	37.5
14. Creating daily menus	28.1
15. Enjoying preparing and eating foods with loved ones	75.0
16. Preparing meals for the week	21.9
17. Eating out less often	40.6
18. Having snacks on a plate	3.3
19. Separating food and work	54.8
20. Eating with others at set times and places	32.3
21. Setting a schedule for eating	48.4
22. Being able to manage time and self in order to suspend other activities and make time for eating	70.0
23. Considering the health value of every item in shopping list	16.1
24. Not going grocery shopping without a list	28.1
25. Not going to the grocery store hungry	37.5
26. Keeping a fruit bowl at home	53.1
27. Keeping healthy snacks at work to prevent selection of unhealthy food	62.5
28. Reading information food panels, labels, and ingredients	59.4
29. Eating without distractions	62.5
30. Having no more than a three hours between meals and snacks	18.8
31. Not skipping meals	59.4
32. Only eating meals and snacks when hungry	48.4
33. Eating when hungry, but not extremely hungry	53.1
34. Tolerating hunger sufficiently to conform to the social structure of meals and snacks	9.4
35. Distinguishing between true hunger and non-hunger triggers	68.8
36. Providing yourself with rewarding meals and snacks	34.4
37. Seeking food rather than avoiding it	56.3
38. Not engaging in behaviors designed to compensate for or eliminate foods eaten	68.8
39. Building relationships with nutritionists	9.4
40. Seeking advice from experts on healthy eating	31.3
41. Enjoying learning to eat new foods	68.8
42. Trying at least one new healthy recipe per week	9.4
43. Eating three meals a day	28.1
44. Managing food context by planning, acquiring, storing, preparing, and providing food	61.3
45. Finding your healthy eating style and managing it for a lifetime	65.6
46. Making small changes to diet at a time	53.1
47. Rejecting dieting	75.0
48. Rejecting food rules	62.5
49. Giving oneself unconditional permission to eat	53.1
*Round 2*	
Focusing on making pleasurable meals	62.5
2. Listening to and connecting with hunger	79.2
3. Eating foods that make your body feel good	70.8
*Round 3*	
Making time for meals	73.9

**Table A6:** Highly rejected survey items in section 3.

Section 3: Thought and Feelings Related to Eating and Food.	% Endorsed
*Round 1*	
Rejecting the dieting mentality	77.4
2. Rejecting conventional food rules	45.2
3. Thinking about the long term consequences of eating poorly	71.0
4. Accepting the body weight that evolves from such internally regulated eating	67.8
5. Giving yourself emotional permission to eat desired food	77.4
6. Being comfortable with what you eat, including food that is high in sugar, salt, and fat	51.6
7. Being confident that there will be enough rewarding food at structured eating times to satisfy hunger and appetite	58.2
8. Being calm in the presence of food, including unfamiliar and disliked food items	67.7
9. Being able to settle for less-preferred food when necessary to satisfy caloric or other nutritional needs	67.7
10. Noticing the effects food has on your figure and feelings	35.5
11. Keeping harmony among food desires, food choices, and amounts eaten	61.3
12. Having responsive attunement to inner and outer food experiences	64.5
13. Considering and appreciating where food comes from	71.0
14. Engaging your senses by noticing colors, smells, sounds, textures, and flavors	70.0
15. Considering every food to be allowed	58.1
16. Following the 80/20 plan: Eat 80% healthy, 20% for splurges	6.5
17. Choosing a diet that is easy to stick to	38.7
18. Considering eating as a pleasurable activity	74.2
19. Not using food as a means of emotional regulation	71.0
20. Using food as a means of honoring your body	66.7
21. Eating foods that are tasty	77.4
22. Not experiencing negative feelings or thoughts (guilt, etc.) regarding foods and eating	77.4
*Round 2*	
Accepting that everyone’s eating experiences are unique	79.2
2. Acknowledging responses to food without judgement	75
3. Focusing on the quality of nutrition instead of counting calorie	75
4. Acknowledging there is no “good” or “bad” food	75
5. Understanding the dignity and importance of eating	70.8
6. Identifying and resolving guilty feelings about food	79.2
7. Paying attention to what you are eating	75
*Round 3*	
Being confident you can trust yourself to manage food and eating	65.2
2. Learning to cope with anxiety and guilt about food	60.8
3. Eating foods that are satisfying	73.9

Note: Rejected items had below 79 percent endorsement rate.
